# Chloroplast Calcium Signaling in the Spotlight

**DOI:** 10.3389/fpls.2020.00186

**Published:** 2020-03-11

**Authors:** Lorella Navazio, Elide Formentin, Laura Cendron, Ildikò Szabò

**Affiliations:** ^1^ Department of Biology, University of Padova, Padova, Italy; ^2^ Botanical Garden, University of Padova, Padova, Italy

**Keywords:** chloroplasts, organellar calcium signaling, calcium-permeable channels, calcium transporters, calcium binding proteins, genetically encoded calcium indicators, chloroplast calcium uniporter

## Abstract

Calcium has long been known to regulate the metabolism of chloroplasts, concerning both light and carbon reactions of photosynthesis, as well as additional non photosynthesis-related processes. In addition to undergo Ca^2+^ regulation, chloroplasts can also influence the overall Ca^2+^ signaling pathways of the plant cell. Compelling evidence indicate that chloroplasts can generate specific stromal Ca^2+^ signals and contribute to the fine tuning of cytoplasmic Ca^2+^ signaling in response to different environmental stimuli. The recent set up of a toolkit of genetically encoded Ca^2+^ indicators, targeted to different chloroplast subcompartments (envelope, stroma, thylakoids) has helped to unravel the participation of chloroplasts in intracellular Ca^2+^ handling in resting conditions and during signal transduction. Intra-chloroplast Ca^2+^ signals have been demonstrated to occur in response to specific environmental stimuli, suggesting a role for these plant-unique organelles in transducing Ca^2+^-mediated stress signals. In this mini-review we present current knowledge of stimulus-specific intra-chloroplast Ca^2+^ transients, as well as recent advances in the identification and characterization of Ca^2+^-permeable channels/transporters localized at chloroplast membranes. In particular, the potential role played by cMCU, a chloroplast-localized member of the mitochondrial calcium uniporter (MCU) family, as component of plant environmental sensing is discussed in detail, taking into account some specific structural features of cMCU. In summary, the recent molecular identification of some players of chloroplast Ca^2+^ signaling has opened new avenues in this rapidly developing field and will hopefully allow a deeper understanding of the role of chloroplasts in shaping physiological responses in plants.

## Introduction

Calcium is a fundamental intracellular messenger involved in a wide range of different signaling pathways in all eukaryotes. In plants, Ca^2+^ has been shown to participate in the transduction of a large variety of environmental stimuli of both abiotic and biotic nature (Dodd et al., 2010). A complex Ca^2+^ homeostatic and signaling machinery allows for a tight regulation of the intracellular concentration of the ion ([Ca^2+^]) and its variations during signal transduction (Kudla et al., 2018). Plant organellar Ca^2+^ signaling is a rapidly expanding field of investigation, also thanks to the increasing availability of novel genetically encoded Ca^2+^ indicators, specifically targeted to different intracellular compartments (Costa et al., 2018). In addition to the vacuole, considered as the main stimulus-releasable Ca^2+^ store in the plant cell, other organelles, *i.e.* chloroplasts, have recently come to the fore. The detection of stimulus-specific intra-chloroplast Ca^2+^ signals in response to different environmental cues has highlighted the contribution of chloroplasts to shaping cytosolic Ca^2+^ signatures. In this mini-review we present the most recent research works dealing with the monitoring of chloroplast Ca^2+^ concentration and its changes during signal transduction events. Moreover, we focus on the recently reported identification and biochemical characterization of some molecular players involved in chloroplast Ca^2+^ handling. Current evidence for a crucial role of chloroplasts as stress sensors and future avenues of investigation in this promising field are also discussed.

## The Emerging Role of Chloroplast Calcium Signaling in the Transduction of Biotic and Abiotic Stress Signals

Chloroplasts have long been known to be involved in intracellular Ca^2+^ homeostasis and signaling. The regulatory role played by these organelles on intracellular Ca^2+^ handling is two-fold: i) a tight control of intra-organellar [Ca^2+^] is essential for the proper functioning of the chloroplast physiology, *e.g.* the regulation of photosynthesis, as well as other chloroplast-localized processes (Stael et al., 2012b; Rocha and Vothknecht, 2012; Nomura and Shiina, 2014; Hochmal et al., 2015); ii) transient changes in stromal [Ca^2+^] ([Ca^2+^]_str_), evoked in response to different stress stimuli, in turn can shape intracellular Ca^2+^ signals, thereby affecting Ca^2+^-mediated signaling circuits.

After the pioneering work conducted by Johnson et al. (Johnson et al., 1995) and Sai and Johnson (Sai and Johnson, 2002), who monitored [Ca^2+^] in the chloroplast stroma by means of a chloroplast-targeted aequorin chimera, precise measurements of Ca^2+^ levels inside the different chloroplast subcompartments have been lacking for a long time. However, in the last few years the increasing availability of specifically targeted Ca^2+^ reporters has rapidly expanded the possibility of accurately monitoring organellar Ca^2+^ dynamics. The set up of a toolkit of aequorin-based probes targeted to the different subcompartments of chloroplasts (outer and inner envelope membranes, stroma, thylakoids) has allowed for the elucidation of stimulus-specific intra-organellar Ca^2+^ signals and their contribution to fine-tuning cytosolic Ca^2+^ signatures (Mehlmer et al., 2012; Sello et al., 2016; Sello et al., 2018). A complementary approach based on the design of a cameleon probe directed to the chloroplast stroma further permitted Ca^2+^ imaging in single chloroplasts, highlighting organelle-autonomous Ca^2+^ transients (Loro et al., 2016). The establishment of aequorin reporters targeted to the thylakoid lumen and thylakoid membrane highlighted the ability of thylakoids to store 3- to 5-fold higher [Ca^2+^] with respect to the stroma (about 500 nM in the thylakoid lumen *versus* 100÷150 nM in the stroma, in resting conditions in the dark), as well as their contribution to the modulation of intra-chloroplast Ca^2+^ signals (Sello et al., 2018).

Chloroplast Ca^2+^ signals have been shown to be triggered by a large number of different stimuli of both biotic and abiotic nature. Elicitors of plant defence responses, such as the fungal-derived protein cryptogein and the plant cell wall-derived pectin fragments oligogalacturonides, were found to evoke transient Ca^2+^ elevations in the chloroplast stroma of *Nicotiana tabacum* and *Arabidopsis thaliana* plant cell suspension cultures (Manzoor et al., 2012; Sello et al., 2018). Moreover, the bacterial flagellin peptide flg22 was demonstrated to trigger a chloroplast Ca^2+^ response in the chloroplast stroma of Arabidopsis rosette leaves, peaking later than the cytosolic Ca^2+^ elevation (Nomura et al., 2012; Nomura and Shiina, 2014). In this latter work, a striking chloroplast-mediated transcriptional reprogramming during plant immune responses was demonstrated, uncovering an unanticipated link between chloroplast and nuclear plant innate immunity *via* ROS and Ca^2+^ signaling (Stael et al., 2015). The calcium-sensing receptor CAS, a thylakoid-localized protein of not yet well-defined function, was found to be involved in the generation of the flg22-induced stromal Ca^2+^ transient and chloroplast-mediated activation of defence gene expression (Nomura et al., 2012).

Different abiotic cues, such as cold, oxidative, salt and osmotic stresses were found to evoke stimulus-specific Ca^2+^ signals in the chloroplast stroma (Nomura et al., 2012; Sello et al., 2016; Sello et al., 2018; Teardo et al, 2019). Whereas these stimuli were shown to activate Ca^2+^ responses in both chloroplasts and non-green plastids (Sello et al., 2016), the light-to-dark transition was found to elicit a chloroplast-specific response (Sello et al., 2016; Loro et al., 2016). Although the precise mechanisms underlying dark-induced chloroplast Ca^2+^ fluxes remain to be unravelled, the circadian gating of dark-induced chloroplast and cytosolic Ca^2+^ elevations has recently been demonstrated (Martí Ruiz et al., 2020), uncovering an intriguing link between eukaryotic circadian clocks and chloroplasts.

In contrast to the above-mentioned stimuli, that have been demonstrated to trigger Ca^2+^ transients in both chloroplasts and the cytosol, increases in absolute temperature were found to evoke Ca^2+^ responses specific to chloroplasts, as no corresponding elevations were detected in the cytosol (Lenzoni and Knight, 2019). Interestingly, also in this case the chloroplast Ca^2+^ response was found to be partially dependent on CAS (Lenzoni and Knight, 2019).

Taken together, the above findings strongly highlight the ability of chloroplasts to perceive and transduce environmental signals in a Ca^2+^-dependent manner. However, compared to the large amount of information progressively cumulating on the generation of chloroplast Ca^2+^ signals, information about Ca^2+^-permeable channels/transporters localized at chloroplast membranes has long lagged behind.

## Current Knowledge of the Molecular Players Involved in Ca^2+^ Handling in Chloroplasts

The extent, duration and frequency (*i.e.* signature) of free Ca^2+^ elevation in the cytosol ([Ca^2+^]_cyt_) acts as a signal to be implemented in the transducing machinery of the cell. Different stimuli are followed by different Ca^2+^ signatures, leading in turn to different specific responses, in terms of gene expression, protein activity and localization. The Ca^2+^ signature is shaped by the activity of Ca^2+^-permeable channels and transporters regulating the ion entry into and exit from the cytosol, respectively. Ca^2+^-permeable channels are grouped in five families: cyclic nucleotide-gated channels (CNGCs), glutamate receptors-like channels (GLRs), two-pore channels (TPCs), mechanosensitive channels (MCAs), hyperosmolality gated channels (OSCAs) (Demidchik et al., 2018). Ca^2+^ transport off the cytosol to restore the resting [Ca^2+^]_cyt_ is mediated by energy-driven pumps/transporters belonging to the P-type ATPases, such as P1B-type calcium/heavy metal cation-transporting ATPase (AtHMA1), P2A-type calcium cation-transporting ATPase (ECAs) and P2B-type calcium cation-transporting ATPase (ACAs) (García Bossi et al., 2020). Other Ca^2+^ transporters are grouped in the CaCA family (CAX-type proton:calcium cation exchanger, CCX-type cation:calcium cation exchanger, MHX-type proton:magnesium cation exchanger, NCL/EF-CAX-type cation exchanger, EF-CAX-type cation exchanger) (Pittman and Hirschi, 2016) and CaCA2 family (PAM71-type manganese/calcium cation transporter).

The transduction of the Ca^2+^ signal is mediated by Ca^2+^ -dependent/binding proteins. The Arabidopsis genome encodes for 250 proteins harbouring at least one Ca^2+^ binding domain (EF-hand), hence acting as putative Ca^2+^ sensors [*e.g.* (Ranty et al., 2016)]. Calmodulins (CaMs), calmodulin-like (CaMLs), calcineurin B-like proteins (CBLs) and Ca^2+^-dependent protein kinases (CPKs) all harbour EF hand motifs. Ca^2+^ sensors directly (CPKs) or indirectly (CaMs, CaMLs, CBLs) [*e.g.* (Sanyal et al., 2015; Kudla et al., 2018)] modulate protein activity (*e.g.* ion channels, metabolic enzymes) and/or protein subcellular localization (*e.g.* transcription factors). The redundancy of sensor isoforms allows the discrimination between different signals and carry the specificity of the message brought by the Ca^2+^ signature.

To our knowledge, Ca^2+^-binding proteins acting as buffers in the chloroplast have not yet been identified. Nevertheless, organellar Ca^2+^ buffering mechanisms are likely to play an essential role, generating heterogeneity in local Ca^2+^ concentrations inside chloroplasts. How Ca^2+^ is stored in the chloroplast remains an open question for future investigations, aimed to unravel whether Ca^2+^ interacts with specific Ca^2+^ binding proteins or with the thylakoid surface, which harbours a significant amount of phosphorylated proteins that have been suggested to bind calcium ions (Rocha and Vothknecht, 2012; Stael et al., 2012a; Stael et al., 2012b).

The major part of research carried out so far has focused on the analysis of the cytosolic Ca^2+^ signature, but the possibility to study Ca^2+^ dynamics in organelles by targeting Ca^2+^ probes to plastids has recently allowed the understanding of the existence of organellar Ca^2+^ transients in response to external stimuli. These findings pose the question of the identity of players involved in shaping and transducing the Ca^2+^ signal coming from organelles. The existence of peculiar and dedicated pathways for Ca^2+^ handling in organelles can be a possibility, and/or the machinery may comprise some already known players that may localize to chloroplasts as well (Finazzi et al., 2015; Pottosin and Shabala, 2015; Carraretto et al., 2016).

Recently, two proteins belonging to the family of the mitochondrial calcium uniporter (MCU) have been found to mediate Ca^2+^ transport across the mitochondria and chloroplast membranes, respectively AtMCU1 (Teardo et al, 2017) and AtMCU6 (later renamed AtcMCU (Teardo et al., 2019). In animal cells the only isoform, MCU (De Stefani et al., 2011; Baughman, 2011) is responsible for Ca^2+^ loading into mitochondria, thus helping recovery of resting [Ca^2+^]_cyt_. New evidence supports the involvement of MCU isoforms in shaping the organellar Ca^2+^ signatures in plants as well (Wagner et al., 2015; Teardo et al., 2017; Selles et al., 2018; Teardo et al., 2019). In particular, cMCU is involved in the generation of the stromal Ca^2+^ transient specific for the osmotic stress and mutants lacking cMCU showed an improved drought tolerance (Stael, 2019; Teardo et al., 2019).

It is now commonly acknowledged that a protein can localize to different cell compartments (Karniely and Pines, 2005), as it has been proven also for proteins involved in Ca^2+^ handling (**Table 1**). AtGLR3.4 and AtGLR3.5, two Ca^2+^ -permeable channels belonging to the GLR family, have a dual localization, at the plasma membrane and chloroplasts the former (Teardo et al., 2010; Teardo et al., 2011), in mitochondria and chloroplasts the latter (Teardo et al., 2015). Both seem to play a role in ABA signaling under abiotic stress (Cheng et al., 2018; Ju et al., 2020), although their direct involvement in organellar Ca^2+^ signaling under abiotic stress has to be investigated more in depth.

**Table 1:** List of proteins involved in Ca^2+^ handling predicted to be located in plastids.

Gene ID	Protein Name	Description	Protein family	Predicted Localization (Aramemnon or SUBA4)	Experimental Localization (FP, MS/MS)	involved in	references
***Ca^2+^ sensors***							
*At1g18890*	AtCPK10	Calcium-dependent protein kinase 10	Calcium Dependent Protein Kinase	plastid, mitochondrion, cytosol, nucleus	nucleus	drought, ABA, stomatal closure	Zou et al., 2010; Liu et al., 2017
*At1g35670*	AtCPK11	Calcium-dependent protein kinase 11	Calcium Dependent Protein Kinase	plastid, mitochondrion, cytosol, nucleus	nucleus, cytosol, PM	pollen tube growth, salt and drought induced, salt and ABA signaling	Urao et al., 1994; Rodriguez Milla et al., 2006; Zhu et al., 2007; Benschop et al., 2007; Ito et al., 2011; Zhao et al., 2013
At2g17890	AtCPK16	Calcium-dependent protein kinase 16	Calcium Dependent Protein Kinase	plastid, mitochondrion, cytosol	PM		Dammann et al., 2003; Stael et al., 2011
At2g31500	AtCPK24	Calcium-dependent protein kinase 24	Calcium Dependent Protein Kinase	plastid, mitochondrion, cytosol, nucleus	nucleus, PM	pollen tube growth	Gutermuth et al., 2013; Zhao et al., 2013
**At2g38910**	AtCPK20	Calcium-dependent protein kinase 20	Calcium Dependent Protein Kinase	plastid, nucleus, membrane	plastid, PM		Dammann et al., 2003; Behrens et al., 2013; Gutermuth et al., 2013
At3g10660	AtCPK2	Calcium-dependent protein kinase 2	Calcium Dependent Protein Kinase	plastid, nucleus, mitochondrion, cytosol	PM		Gutermuth et al., 2013
**At4g04695**	AtCPK31	Calcium-dependent protein kinase 31	Calcium Dependent Protein Kinase	nucleus, plastid, cytosol, mitochondrion	plastid, PM	arsenite uptake	Helm et al., 2014; Ji et al., 2017
At4g04720	AtCPK21	Calcium-dependent protein kinase 21	Calcium Dependent Protein Kinase	PM, cytosol, mitochondrion, plastid, nucleus	PM	interacts with SLAC1, ABI1, SLAH3, GORK	Dammann et al., 2003; Alexandersson et al., 2004; Nelson et al., 2006; Benschop et al., 2007; Marmagne et al., 2007; Mitra et al., 2009; Keinath et al., 2010; Geiger et al., 2010; Zhang and Peck, 2011; Elmore et al., 2012; Nikolovski et al., 2012; Bernfur et al., 2013; Demir et al., 2013; Zargar et al., 2015; De Michele et al., 2016; van Kleeff et al., 2018
*At4g09570*	AtCPK4	Calcium-dependent protein kinase 4	Calcium Dependent Protein Kinase	cytosol, nucleus, mitochondrion, plastid	PM, cytosol, nucleus	ABA and salt response; interacts with plastid proteins	Dammann et al., 2003; Zhu et al., 2007; Mitra et al., 2009; Uno et al., 2009; Ito et al., 2011; Li et al., 2018
At4g21940	AtCPK15	Calcium-dependent protein kinase 15	Calcium Dependent Protein Kinase	cytosol, plastid, nucleus, mitochondrion	PM		Li et al., 2012; Bernfur et al., 2013
At4g23650	AtCPK3	Calcium-dependent protein kinase 3	Calcium Dependent Protein Kinase	plastid, mitochondrion, cytosol, nucleus	cytosol, nucleus, PM, Golgi, tonoplast	stomatal closure	Dammann et al., 2003; Alexandersson et al., 2004; Dunkley et al., 2006; Mori et al., 2006; Nelson et al., 2006; Benschop et al., 2007; Mitra et al., 2009; Keinath et al., 2010; Ito et al., 2011; Elmore et al., 2012; Li et al., 2012; Nikolovski et al., 2012; Latz et al., 2013; Zargar et al., 2015; Heard et al., 2015; De Michele et al., 2016
At4g36070	AtCPK18	Calcium-dependent protein kinase 18	Calcium Dependent Protein Kinase	plastid, mitochondrion, peroxisome, PM			
*At5g04870*	AtCPK1/AtAK1	Calcium-dependent protein kinase 1	Calcium Dependent Protein Kinase	plastid, nucleus, cytosol, mitochondrion	peroxisome, MVB, cytosol, PM	salt and drought	Dammann et al., 2003; Chen et al., 2010; Drakakaki et al., 2012; De Michele et al., 2016; Huang et al., 2018
At5g12180	AtCPK17	Calcium-dependent protein kinase 17	Calcium Dependent Protein Kinase	cytosol, nucleus, mitochondrion, plastid	PM	pollen tube tip growth	Myers et al., 2009; Gutermuth et al., 2013; Bernfur et al., 2013
At5g12480	AtCPK7	Calcium-dependent protein kinase 7	Calcium Dependent Protein Kinase	plastid, mitochondrion, cytosol, nucleus	PM, Golgi	root hydraulic conductivity	Dammann et al., 2003; Marmagne et al., 2007; Benschop et al., 2007; Elmore et al., 2012; Heard et al., 2015; Li et al., 2015
At5g19360	AtCPK34	Calcium-dependent protein kinase 34	Calcium Dependent Protein Kinase	cytosol, nucleus, mitochondrion, plastid	PM	pollen tube tip growth	Myers et al., 2009; Gutermuth et al., 2013; Bernfur et al., 2013
At5g19450	AtCPK8	Calcium-dependent protein kinase 8	Calcium Dependent Protein Kinase	cytosol, nucleus, mitochondrion, plastid	PM	ABA signaling and H_2_O_2_ homeostasis in guard cells	Dammann et al., 2003; Nühse et al., 2003; Nühse et al., 2004; Benschop et al., 2007; Chen et al., 2010; Keinath et al., 2010; Zhang and Peck, 2011; Elmore et al., 2012; Zargar et al., 2015; Zou et al., 2015
At5g24430	AtCRK4	Calcium-dependent protein kinase 4	Calcium Dependent Protein Kinase	plastid, nucleus, cytosol, mitochondrion	PM		Benschop et al., 2007; Marmagne et al., 2007; Chen et al., 2010; Keinath et al., 2010; Zhang and Peck, 2011; Li et al., 2012; Szymanski et al., 2015; De Michele et al., 2016
*At5g66210*	AtCPK28	Calcium-dependent protein kinase 28	Calcium Dependent Protein Kinase	cytosol, plastid, mitochondrion, nucleus	PM	plant immunity	Dammann et al., 2003; Benschop et al., 2007; Elmore et al., 2012; Monaghan et al., 2014; Monaghan et al., 2015; Matschi et al., 2015; De Michele et al., 2016
At2g15680	AtCML30	Calmodulin-like protein 30	Calmodulin-like protein	plastid, mitochondrion, cytosol, PM	mitochondrion		Chigri et al., 2012
At2g41410	AtCML35	Probable calcium-binding protein CML35	Calmodulin-like protein	plastid, mitochondrion, nucleus, cytosol, PM	PM, vacuole	dark induced	Lee et al., 2005; Benschop et al., 2007; Whiteman et al., 2008; Elmore et al., 2012; Li et al., 2012; De Michele et al., 2016
At2g43290	AtCML5	Calmodulin-like protein 5	Calmodulin-like protein	plastid, mitochondrion, nucleus, cytosol, PM, ER, extracellular	ER, Golgi	dark and touch induced	Lee et al., 2005; Ruge et al., 2016
At3g10190	AtCML36	Calmodulin-like protein 36	Calmodulin-like protein	plastid, nucleus, mitochondrion, cytosol	PM	ACA8 activation	Benschop et al., 2007; Astegno et al., 2017
At3g29000	AtCML45	Calmodulin-like protein 45	Calmodulin-like protein	plastid, mitochondrion, Golgi, cytosol, PM, ER			
At3g50770	AtCML41	Probable calcium-binding protein CML41	Calmodulin-like protein	plastid, mitochondrion, cytosol			
At4g26470	AtCML21	Calmodulin-like protein 21		cytosol, PM, mitochondrion, nucleus, plastid	cell wall		Nguyen-Kim et al., 2016
At5g04170	AtCML50	Probable calcium-binding protein CML50	Calmodulin-like protein	plastid, extracellular space, ER, mitochondrion, PM, nucleus	cell wall		Nguyen-Kim et al., 2016
At5g39670	AtCML46	Calmodulin-like protein 46	Calmodulin-like protein	cytosol, plastid, mitochondrion, ER, Golgi, nucleus, extraellular			
*At5g42380*	AtCML37	Calcium-binding protein CML37	Calmodulin-like protein	plastid, nucleus, cytosol, PM,mitochondrion	cytosol, nucleus	drought, wounding	Vanderbeld and Snedden, 2007; Inzè et al., 2012; Scholz et al., 2014; Scholz et al., 2015
At4g32060	AtMICU	Calcium uptake protein, mitochondrial		PM, mitochondrion, plastid	mitochondrion	regulation of Ca^2+^ uniporters (MCUs)	Wagner et al., 2015; Teardo et al., 2017
*At4g33000*	AtCBL10	Calcineurin B-like protein 10	Calcineurin B-like protein	plastid, mitochondrion, PM, ER	PM, tonoplast	salt tolerance	Mitra et al., 2009; Ma et al., 2019; Yang et al., 2019
***At5g23060***	AtCAS	Calcium sensing receptor	Calcium sensing receptor	plastid, mitochondrion	plastid, thylakoid, Golgi, mitochondrion, nucleus	high light, stomatal regulation, drought tolerance	Vainonen et al., 2008; Weinl et al., 2008; Behrens et al., 2013; Helm et al., 2014; Tomizioli et al., 2014; Wang et al., 2014; Heard et al., 2015; Fakih et al., 2016; Fromm et al., 2016; Melonek et al., 2016; Senkler et al., 2017; Cutolo et al., 2019
***Ca^2+^ transporters/channels***							
*At1g53210*	AtNCL	Sodium/calcium exchanger	NCL/EF-CAX-type cation exchanger	plastid, mitochondrion, Golgi, cytosol, PM, ER	PM, tonoplast	flowering time, auxin signaling, salt stress	Nikolovski et al., 2012; Elmore et al., 2012; Li et al., 2016; Wang et al., 2012; Yoshida et al., 2013; Szymanski et al., 2015; Zargar et al., 2015; Li et al., 2016
At2g34020		Putative EF-CAX-type cation exchanger	EF-CAX-type cation exchanger	PM, plastid, mitochondrion, ER, Golgi			
*At2g38170*	AtCAX1	High-affinity calcium/proton cation exchanger	CAX-type proton:calcium cation exchanger	plastid, mitochondrion, Golgi, PM, tonoplast	tonoplast	Cd^2+^ tolerance; pH regulation; hormone signaling; guard cell dynamics; stress response	Cheng et al., 2003; Conn et al., 2011; Cho et al., 2012; Baliardini et al., 2015; Hocking et al., 2017
At3g14070	AtCCX3/CAX9	Cation/calcium exchanger 3	CCX-type cation:calcium cation exchanger	plastid, mitochondrion, Golgi, PM, ER	endomembrane		Morris et al., 2008
At3g51860	AtCAX3	High-affinity calcium/proton cation exchanger	CAX-type proton:calcium cation exchanger	plastid, mitochondrion, Golgi, PM, tonoplast	tonoplast	pH regulation; hormone signaling; guard cell dynamics	Manohar et al., 2011; Cho et al., 2012; Hocking et al., 2017
At5g01490	AtCAX4	High-affinity calcium/proton cation exchanger	CAX-type proton:calcium cation exchanger	plastid, ER, PM, tonoplast	tonoplast	Cd^2+^ accumulation	Cheng et al., 2002; Mei et al., 2009
At2g23790	AtMCU3	Putative channel component of MCUC calcium uniporter complex	Component of MCU calcium uniporter complex	plastid, mitochondrion, nucleus	tonoplast		Yoshida et al., 2013
At4g36820	AtMCU4	Putative channel component of MCUC calcium uniporter complex	Component of MCU calcium uniporter complex	mitochondrion, chloroplast, nucleus	mitochondrion		Teardo et al., 2017
***At5g66650***	AtMCU6/AtcMCU	Putative channel component of MCUC calcium uniporter complex	Component of MCU calcium uniporter complex	plastid, mitochondrion	plastid, mitochondrion	drought, hypoxia	Teardo et al., 2019; Lee and Bailey-Serres, 2019
***At1g05200***	AtGLR3.4	Putative GLR-type amino acid-gated calcium cation channel	GLR-type ligand-gated cation channel	PM, plastid, ER, Golgi, mitochondrion	plastid, PM	Ca^2+^ transport; salt and cold stress; ABA signaling; seed germination; lateral root development	Meyerhoff et al., 2005; Stephens et al., 2008; Teardo et al., 2011; Vincill et al., 2013; Cheng et al., 2018
At2g17260	AtGLR3.1	Putative GLR-type calcium cation-permeable channel	GLR-type ligand-gated cation channel	PM, plastid, ER, Golgi	endomembrane	stomatal closure	Cho et al., 2009; Kong et al., 2016; Nguyen et al., 2018a
***At2g32390***	AtGLR3.5	Putative GLR-type calcium cation-permeable channel	GLR-type ligand-gated cation channel	PM, plastid, mitochondrion, nucleus	mitochondrion, plastid	Ca^2+^ transport; ABA signaling; seed germination; stomatal closure	Teardo et al., 2015; Kong et al., 2016; Ju et al., 2020
At5g11210	AtGLR2.5	Putative GLR-type calcium cation-permeable channel	GLR-type ligand-gated cation channel	plastid, mitochondrion, PM	PM		Mitra et al., 2009
At1g69450	AtOSCA2.4	Early-responsive to dehydration stress protein (ERD4)	OSCA1/2/3-type Ca^2+^-permeable hyperosmolality-gated channel	chloroplast, mitochondrion, PM, Golgi	PM		Yuan et al., 2014
**At3g54510**	AtOSCA2.5	Hyperosmolality-gated calcium-permeable channel	OSCA1/2/3-type Ca^2+^-permeable hyperosmolality-gated channel	mitochondrion, plastid, nucleus, Golgi,ER, PM	ER, mitochondrion, plastid		Lee et al., 2011
At4g02900	AtOSCA1.7	Hyperosmolality-gated calcium-permeable channel	OSCA1/2/3-type Ca^2+^-permeable hyperosmolality-gated channel	mitochondrion, plastid, nucleus, Golgi,ER, PM			
At4g35870	AtOSCA4.1/AtGFS10	Calcium-permeable channel-like protein	OSCA4-type unspecified channel	chloroplast, mitochondrion, PM, Golgi, nucleus	Golgi		Heard et al., 2015
**At4g37270**	AtHMA1	Thapsigargin-sensitive calcium/heavy metal cation-transporting P1B-type ATPase	P1B-type heavy metal cation-transporting ATPase	plastid, mitochondrion, PM	chloroplast envelope	photosynthesis	Seigneurin-Berny et al., 2006; Higuchi et al., 2009; Ferro et al., 2010; Nikolovski et al., 2012; Tomizioli et al., 2014
**At1g27770**	AtACA1	Calcium-transporting ATPase	P2B-type calcium cation-transporting ATPase	plasma membrane, plastid, cytosol, ER, mitochondrion, nucleus	plastid, ER, PM, tonoplast, microtubule		Huang et al., 1993; Dunkley et al., 2006; Benschop et al., 2007; Mitra et al., 2009; Zhang and Peck, 2011; Yoshida et al., 2013; Hamada et al., 2013
**At3g21180**	AtACA9	Calcium-transporting ATPase	P2B-type calcium cation-transporting ATPase	plasma membrane, plastid, cytosol, ER, mitochondrion, nucleus	plasma membrane, plastid, cytosol	pollen development,	Schiott et al., 2004; Tomizioli et al., 2014
*At4g37640*	AtACA2	Calcium-transporting ATPase	P2B-type calcium cation-transporting ATPase	PM, ER, plastid, mitochondrion, vacuole	Golgi, ER, PM	salt tolerance in yeast	Dunkley et al., 2006; Benschop et al., 2007; Anil et al., 2008; Zhang and Peck, 2011; Nikolovski et al., 2012; Heard et al., 2015
**At5g53010**		Calcium-transporting ATPase, putative	P2B-type calcium cation-transporting ATPase	mitochondrion, PM, ER	plastid		Tomizioli et al., 2014
**At1g64150**	AtBICAT1/AtPAM71/AtCCHA1	Putative calcium/manganese cation transporter	PAM71-type manganese/calcium cation transporter	plastid, mitochondrion	thylakoid membrane	Mn^2+^ homeostasis, phototropic growth, chloroplast Ca^2+^ homeostasis, photosynthesis	Wang et al., 2016; Schneider et al., 2016; Frank et al., 2019
**At4g13590**	AtBICAT2/AtCMT1	Putative calcium/manganese cation transporter	PAM71-type manganese/calcium cation transporter	plastid, mitochondrion	chloroplast envelope	Mn^2+^ homeostasis, phototropic growth, chloroplast Ca^2+^ homeostasis, photosynthesis	Ferro et al., 2010; Zybailov et al., 2008; Ferro et al., 2010; Tomizioli et al., 2014; Eisenhut et al., 2018; Zhang et al., 2018; Frank et al., 2019
***Others***							
**At1g64850**			Calcium-binding EF hand family protein	vacuole, mitochondrion, plastid, nucleus, vacuole	plastid, peroxisome		Reumann et al., 2009; Ferro et al., 2010; Nikolovski et al., 2012
*At2g42590*	AtGRF9	14-3-3-like protein GF14 mu	14-3-3 protein	nucleus, cytosol, mitochondrion, PM	cytosol, plastid, vacuole, nucleus, PM, peroxisome, Golgi	root growth in water stress, leaf development,cold stress	Mayfield et al., 2012; He et al., 2015; Liu et al., 2017; Omidbakhshfard et al., 2018
At4g08810	AtSUB1		Calcium binding protein	plastid, nucleus, ER, Golgi,	Golgi	cryptochrome and phytochrome coaction	Guo et al., 2001; Parsons et al., 2012
At4g34070			Calcium-binding EF-hand family protein	plastid, mitochondrion, Golgi, ER, cytosol, extracellular			
At4g38810			Calcium-binding EF-hand family protein	plastid, nucleus, mitochondrion, cytosol			

The experimental determined localization comes from MS/MS analyses or fluorescent protein fusion (FP). Articles referring to the original data are reported. In bold proteins proved to be located in chloroplasts. In italics genes involved in stress response. PM, plasma membrane; ER, endoplasmic reticulum.

Querying the protein databases Uniprot (The UniProt Consortium, 2019), SUBA4 (Hooper et al., 2017) and Aramemnon (Schwacke et al., 2003) for *A. thaliana* records with plastidial localization and using “calcium” as keyword, 682 hits can be found in SUBA4, only 43 in Aramemnon and 42 in Uniprot. **Table 1** shows all those proteins belonging to the above-mentioned classes of channels/transporters, sensors and kinases involved in Ca^2+^ signature formation and signaling, whose plastidial localization has been predicted or demonstrated by MS/MS or by fusion to fluorescent proteins (FP).

23 out of 47 proteins belong to Ca^2+^ channels/transporters: 6 are confirmed to be located in plastid membranes either by biochemical and cell biology methods or by mass spectrometry. Among them, for AtcMCU, AtGLR3.4 and AtGLR3.5 a role in stress response was suggested. Altogether, these channels/transporters can be involved in the formation of the plastidial Ca^2+^ transients, along with the putative calcium-transporting protein PAM71/BICAT (Frank et al., 2019). However, this latter protein seems to play a prevalent role in manganese homeostasis rather than in calcium homeostasis (Schneider et al., 2016; Zhang et al., 2018). In addition to Ca^2+^ channels and transporters, Ca^2+^ sensors, namely 21 proteins, are predicted to be located in plastids. However, only three have been confirmed so far: AtCPK20, AtCPK31, and AtCAS. It is worth to mention that CPK20, besides the plastidial localization that was confirmed by MS/MS approaches (Behrens et al., 2013), showed a plasma membrane localization when fused to reporter genes or co-expressed with other CPK members (Gutermuth et al., 2013). CPK31 has also been shown to localize at the plasma membrane when interacting with the arsenite transporter NIP1;1 (Ji et al., 2017). In addition, localization of many CPKs with chloroplast-targeting sequence can be affected by N-acylation. For example, AtCPK20 and 31 are located in the chloroplast, only if its N-acylation is prevented (Stael et al., 2011). Interestingly, AtGRF9, a Ca^2+^-regulated 14-3-3 protein, although not predicted to be located in chloroplasts, has been demonstrated to be present in many compartments, including plastids. This regulatory protein is involved in root and leaf development under water stress (He et al., 2015) and leaf development in general (Omidbakhshfard et al., 2018), but its role in chloroplasts has not yet been explored.

The presence of members of protein families involved in Ca^2+^ transport/sensing supports the idea of a core-machinery determining the observed Ca^2+^ transients in the chloroplast stroma, and putatively in the thylakoid lumen as well. Ca^2+^ sensors are indeed present in plastids, although their activity in deciphering organellar Ca^2+^ signatures has not been fully demonstrated so far. Nevertheless, a recent work points to CAS as mediator of light response and photoacclimation (Cutolo et al., 2019).

The multiple localizations shown by some proteins in **Table 1** awaits further investigation. Recent evidence is pointing to the hypothesis of an inter-connection between organelles and nucleus for material exchanging or signal propagation (Kmiecik et al., 2016). The presence of the Ca^2+^ handling machinery in multiple positions can be part of the retrograde signaling in response to adverse environmental conditions (Pornsiriwong et al., 2017).

## Structural and Functional Comparison Between MCU Isoforms From Different Organisms and the Chloroplast-Localized Homologue in Plants

As mentioned above, AtcMCU is one of the very few molecular entities among the plastidial Ca^2+^ channels/transporters shown to work as a Ca^2+^-permeable ion channel, to mediate indeed Ca^2+^ flux across chloroplast envelope and to participate in the drought stress response in Arabidopsis. While many organisms have only one MCU isoform (Bick et al., 2012), Arabidopsis harbours 6 different isoforms: 5 with clear predicted subcellular localization to mitochondria, whereas AtMCU6/At5g66650 has a predicted localization to either chloroplasts and/or to mitochondria. Localization prediction was confirmed for AtMCU1/At1g09575 (Teardo et al., 2017), AtMCU2/At1g57610 (Wagner et al., 2015; Selles et al., 2018), AtMCU3/At2g23790 (Carraretto et al., 2016). For AtMCU6 an interesting situation was observed: in tissues harbouring mature chloroplasts, AtMCU6 was efficiently targeted to these photosynthetic organelles, whereas in roots the protein was found in mitochondria (Teardo et al., 2019). Thus, either plastid-specific partners promote targeting of AtMCU6/AtcMCU or targeting depends on the metabolic state of a given cell. However, among the possible partners (https://string-db.org/network/3702.AT5G66650.1) no proteins with unique localization to chloroplasts are present. Thus, the mechanism by which dual localization occurs awaits clarification.

The N-terminal domain (NTD) of AtcMCU harbours motifs rich in acidic residues, one of which (107-118) playing a role in Ca^2+^ uptake by cMCU, as demonstrated by mutagenesis studies (D107A/E118K mutant) and Ca^2+^ uptake assays in an aequorin-based *E. coli* system (Teardo et al., 2019). Two groups independently set up the same system to study MCU activity, namely that exploiting *E. coli* stably expressing aequorin (Teardo et al., 2019) or the fluorescent Ca^2+^ reporter GCaMP2 (Fan et al., 2018). This valuable tool allows a quick screening of the effect of MCU residues' mutations and of chemical modulators on the Ca^2+^ flux-mediating activity and may become a method of choice for further structure-function studies.

**Figure 1:**
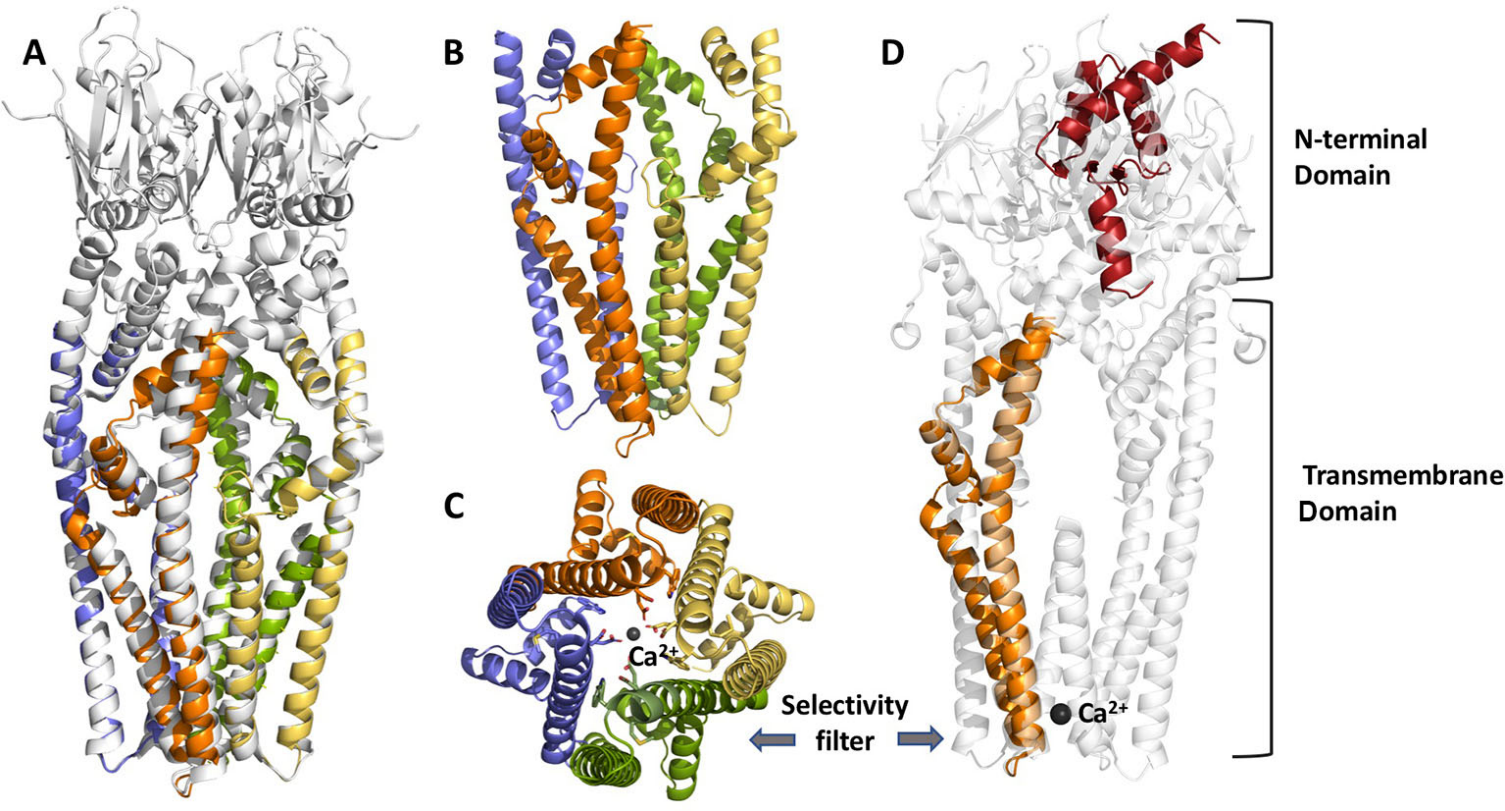
Structural features of chloroplast MCU from *Arabidopsis thaliana*, modelled by Phyre V 2.0. From the left to the right: (panel **A**) cartoon view of the superposition of MCU structure from *Neurospora crassa* (cryoEM, 3.7 Å resolution, PDB:6DT0, grey), used as a reference, and predicted *A. thaliana* cMCU transmembrane tetrameric assembly (coloured chains); (panel **B, C**) details of the transmembrane (TM) and coiled-coil domain (CCD) tetrameric assembly and selectivity filter (panel **C**), where the four chains are shown in yellow, orange, pale violet and green. The key acidic residues within the highly conserved motif (WDXXEP, where X is any hydrophobic residue) of cMCU are highlighted in sticks, as well as the coordinated calcium ion, shown as dark grey sphere; (panel **D**) superposition of the model of one monomer of *A. thaliana* cMCU channel (orange and red) and *N. crassa* MCU tetramers (light grey); cMCU model shown here includes the transmembrane domain (TM), part of the coil-coiled region and the N-terminal domain (NTD), the last predicted according to our previous homology searches and its similarity toward *Dictyostelium discoideum* NTD (PDB:5Z2H, doi: https:/doi.org/10.1101/848002). The superposition underlines the divergence from metazoan NTDs and other structure-known fungal homologues such as NcMCU, CeMCU, MaMCU, and NfMCU.

One common feature of MCU homologs from fungi and Arabidopsis is that they can function as Ca^2+^-permeable channels on their own in contrast to vertebrates, where the uniporter is a complex (MCUC) consisting of multiple subunits, including: 1) the channel forming unit (MCU) with two transmembrane segments and a conserved DXXE sequence forming the Ca^2+^ selectivity filter (see **Figure 1**); 2) regulatory EF-hand proteins MICU1-3; 3) a small, single-pass transmembrane protein, EMRE (Essential MCU REgulator) [for review see *e.g.*(Wagner et al., 2016)]. The structure of MCU homologs from various organisms has been recently solved: 1) from *Fusarium graminearum* and *Metarhizium acridum* revealing a dimer assembly of MCU (Fan et al., 2018); 2) from *Neurospora crassa* (Yoo et al., 2018); 3) from *Neosartorya fischeri* (Nguyen et al., 2018b); and from 4) zebrafish and *Cyphellophora europaea* (Baradaran et al., 2018). All these homologues share high sequence similarity in their transmembrane domains, show a similar pore architecture and a high structural similarity of the NTDs (despite relatively low sequence homology). The amino acid sequence is more similar between Arabidopsis and *Dictyostelium discoideum* than between AtMCUs and human MCU (Teardo et al., 2017). This similarity apparently translates also to the tertiary structure of the two proteins, at least regarding the N-terminal domain, whose structure has been recently resolved for Dictyostelium MCU, proving its divergent evolution (doi: https://doi.org/10.1101/848002) (see **Figure 1**).

In plants and fungi, the pore-forming unit MCU alone is able to allow Ca^2+^ flux, without the need of EMRE, as confirmed by different groups (Tsai et al., 2016; Teardo et al., 2017; Fan et al., 2018; Teardo et al., 2019). In fact, homologs of EMRE are not present in these organisms. The cryo-EM structure of the human MCU-EMRE complex (Wang et al., 2019) suggests that NTD mediates the dimerization of two human MCU tetramers, thereby modulating the function of the channel [although deletion of NTD does not affect Ca^2+^ flux (Lee et al., 2015)]. In contrast to other MCUs, an (R/K)/Q/(R/K/D)/K/L motif is found in the L2 (Oxenoid et al., 2016) (now called CC2a for coiled-coiled domain 2a) (Wang et al., 2019) region of Arabidopsis, Dictyostelium and NfMCU (Teardo et al., 2017; Wang et al., 2019), all being able to form functional MCU without EMRE. It has been proposed that the extended side chain of HsMCU R297 (missing in the above MCUs) on CC2a connects the gate-forming juxtamembrane loop (JML) of MCU to EMRE by forming hydrogen bonds with the hydroxyl group of highly conserved T285 (on the JML of MCU) and a valine residue of EMRE. Interaction between CC2a and EMRE has been proposed as a crucial factor determining the conductivity of the channel formed by MCU tetramers. On the other hand, in the EMRE-independent Dictyostelium MCU, deletion of either CC1 or CC2 caused the loss of function of MCU (Yamamoto et al., 2019), suggesting that these two domains are crucial for MCU function independently of their ability to bind EMRE. Altogether, determination of structural differences among various MCUs accounting for the requirement of EMRE for channel function requires further work.

## Conclusions and Perspectives

In these last few years there has been a surge of papers on Ca^2+^ signaling in chloroplasts, witnessing the crucial role increasingly attributed to these plant-unique organelles in the orchestration of the complex Ca^2+^ signaling network of the plant cell. We foresee that the newly available experimental tools to investigate the role of thylakoids in Ca^2+^-mediated signal transduction, the molecular identification of Ca^2+^ channels/transporters in chloroplast membranes and the determination of the structure of transmembrane proteins by cryo-EM will lead to a rapid development of this exciting field of plant research. Future plant organellar Ca^2+^ signaling studies should also focus on non-photosynthetic plastids, which have recently been proposed to trigger tissue-specific signaling involved in mounting plant systemic stress response (Beltran et al., 2018). Furthermore, the potential interplay of chloroplasts with other intracellular Ca^2+^-mobilizable stores should also be taken into consideration, in view of the well-known structural and functional interactions established by plastids with other organelles (Mathur et al., 2012).

## Author Contributions

LN, EF, and IS jointly contributed to the writing of this manuscript. LC designed the structural model of cMCU presented in **Figure 1**. All authors reviewed and approved the final version of the submitted manuscript.

## Funding

This work was supported by HFSP RG0052 to IS and the University of Padova (PRID 2018, BIRD180317) to LN.

## Conflict of Interest

The authors declare that the research was conducted in the absence of any commercial or financial relationships that could be construed as a potential conflict of interest.
